# Biological Process Linkage Networks

**DOI:** 10.1371/journal.pone.0005313

**Published:** 2009-04-23

**Authors:** Dikla Dotan-Cohen, Stan Letovsky, Avraham A. Melkman, Simon Kasif

**Affiliations:** 1 Department of Computer Science, Ben-Gurion University, Beer Sheva, Israel; 2 Codon Devices, Cambridge, Massachusetts, United States of America; 3 Department of Biomedical Engineering, Boston University, Boston, Massachusetts, United States of America; 4 Bioinformatics Program, Boston University, Boston, Massachusetts, United States of America; 5 Center for Advanced Genomic Technology, Boston University, Boston, Massachusetts, United States of America; 6 Children's Hospital Boston, Harvard/MIT Program in Health Sciences and Technology, Boston, Massachusetts, United States of America; Texas A&M University, United States of America

## Abstract

**Background:**

The traditional approach to studying complex biological networks is based on the identification of interactions between internal components of signaling or metabolic pathways. By comparison, little is known about interactions between higher order biological systems, such as biological pathways and processes.

We propose a methodology for gleaning patterns of interactions between biological processes by analyzing protein-protein interactions, transcriptional co-expression and genetic interactions. At the heart of the methodology are the concept of *Linked Processes* and the resultant network of biological processes, the *Process Linkage Network (PLN)*.

**Results:**

We construct, catalogue, and analyze different types of PLNs derived from different data sources and different species. When applied to the Gene Ontology, many of the resulting links connect processes that are distant from each other in the hierarchy, even though the connection makes eminent sense biologically. Some others, however, carry an element of surprise and may reflect mechanisms that are unique to the organism under investigation. In this aspect our method complements the link structure between processes inherent in the Gene Ontology, which by its very nature is species-independent.

As a practical application of the linkage of processes we demonstrate that it can be effectively used in protein function prediction, having the power to increase both the coverage and the accuracy of predictions, when carefully integrated into prediction methods.

**Conclusions:**

Our approach constitutes a promising new direction towards understanding the higher levels of organization of the cell as a system which should help current efforts to re-engineer ontologies and improve our ability to predict which proteins are involved in specific biological processes.

## Introduction

The study of biological systems at different levels of organization is a rapidly emerging area of computational biology. The majority of research in this field has focused on partitioning genes into biological pathways or processes [1]–[8]. The next hurdle in moving towards the goal of understanding the cell at a systems level is to determine how these partitioned cellular processes work together to achieve the cell's objectives.

With the aim of helping to decipher this higher order connectivity we propose a new methodology for gleaning patterns of interaction between biological processes, manifested by a significantly enriched web of protein-protein interactions, transcriptional coordination or genetic interactions.

At the heart of the methodology described in the paper are the concept of *Linked Processes* and the resultant new network of biological processes, the *Process Linkage Network (PLN)*, whose nodes correspond to biological process terms in the Gene Ontology (GO) database. Using this methodology and exploiting various experimental data and annotations, we are able to uncover different interactive and cooperative relationships between processes. Many of these linked terms are distant from each other in the GO-hierarchy, suggesting perhaps a need to revisit the philosophy to organize biological data as a single taxonomy.

We construct and analyze different types of PLNs based on physical protein-protein interactions (*PPI-PLN*), transcriptional co-expression (*expression-PLN*) and genetic interactions (*GI-PLN*). An analysis of the different PLNs yields intriguing findings: Many of the processes that were found to be linked in the various networks are consistent with biological knowledge, while other links are suggestive of further research to elucidate their very existence and meaning. The process “protein ubiquitination” (GO:0016567), for example, is predicted to be PPI-linked to processes related to protein catabolism while it is expression-linked to processes related to rRNA-processing and GI-linked mainly to cell cycle related processes. A biological explanation for these links is offered in the Results section. More generally, many links connect processes that appear unrelated to each other when only the GO hierarchy is considered, even though the connection fits well with current biological knowledge. This may be attributed in part to the fact that the postulated links are generated for a specific organism whereas the Gene Ontology is meant to be universal, Therefore, these links enrich and complement the relations between processes inherent in the GO hierarchy.

Prediction of functional annotation for proteins is another area in which the knowledge obtained from our new methodology provides potential benefits. The relations between the GO-processes derived from the ontology are utilized in the functional prediction methods, hence it is only natural to utilize in addition the links between processes found by our method. We show that such a careful integration of the links into functional prediction methods increases both the coverage and the accuracy of the methods.

As a further application of PPI-links and expression-links between processes we show that the probability of two *S. cerevisiae* genes to genetically interact is significantly increased once it is known that the two genes participate in PPI-linked processes or expression-linked processes.

This multi-scale perspective on biological networks, examining relationships between the elementary parts as well as “modules” in the form of biological processes suggests a promising new direction for developing a deeper insight into biological function.

## Results

### PPI-Process Linkage Network for the yeast *S. cerevisiae*

We initiated our study by using protein-protein interaction data to identify links between processes in *S. cerevisiae*. Process *a* is called *PPI-linked* to process *b* if the number of proteins belonging to process *b* that interact with proteins belonging to process *a* is considerably larger than expected by chance (measured by p-value). The prediction method and the statistical significance procedures are described in the Methods section and the Supplement (Text S1 and Figure S1). A new network, named Process-Linkage-Network (PLN) is then constructed. In this directional network, nodes represent process-terms and there is an edge from node *a* to node *b*, if process *a* is found to be linked to process *b*. The PPI-PLN constructed for *S. cerevisiae* contains 21,097 edges (links) connecting 1,161 processes (nodes), at a p-value of 0.001. The corresponding q-value (using the FDR correction) is 0.06. The characteristics of this network are given in the Supplement (Text S2 and Figure S2).

In order to get a feel for relative locations in the GO hierarchy, of the terms that a link connects, we employ the semantic similarity measure proposed by Lin [9]. This similarity measure takes on values between 0 and 1 (see Methods section), with the similarity between two terms being 0 if their only common ancestor in the GO-hierarchy is the hierarchy root term: “biological process” (GO:0008150).

Many of the predicted pairs of PPI-linked processes fit well with extant knowledge of cellular function. Some of these pairs link processes that are very similar biologically, in spite of their having very little semantic similarity, meaning that they are distant from each other in the GO structure.

Consider the link between the processes “response to DNA damage stimulus” (GO:0006974) and “chromatin modification” (GO:0016568), as one example. This link reflects the fact that chromatin and histone modification is utilized in the DNA damage response pathway [10]. However, the common ancestor of these two term processes is the hierarchy root term, therefore their semantic similarity is 0.

Fifteen additional examples of PPI-linked processes are given in Table 1. Many of these linked terms are not similar semantically, although the link between them makes perfect sense biologically.

**Table 1:** Fifteen examples of PPI-linked processes.

*Term a*		*Term b*		*Semantic similarity*	*Linkage types*
GO:0006313	DNA transposition	GO:0006974	response to DNA damage stimulus	0.00	PPI, GI, Exp
GO:0006270	DNA replication initiation	GO:0045814	negative regulation of gene expression, epigenetic	0.00	PPI, GI
GO:0051318	G1 phase	GO:0042255	ribosome assembly	0.07	PPI, Exp
GO:0006374	nuclear mRNA splicing via U2-type spliceosome	GO:0007046	ribosome biogenesis	0.07	PPI, Exp
GO:0048311	mitochondrion distribution	GO:0007114	cell budding	0.07	PPI, GI
GO:0006944	membrane fusion	GO:0006887	exocytosis	0.07	PPI, GI
GO:0006360	transcription from RNA polymerase I promoter	GO:0007046	ribosome biogenesis	0.08	PPI, Exp
GO:0006333	chromatin assembly or disassembly	GO:0000087	M phase of mitotic cell cycle	0.09	PPI, GI
GO:0006445	regulation of translation	GO:0006402	mRNA catabolism	0.20	PPI, GI
GO:0042257	ribosomal subunit assembly	GO:0016072	rRNA metabolism	0.24	PPI, GI, Exp
GO:0000282	bud site selection	GO:0042273	ribosomal large subunit biogenesis	0.27	PPI, Exp
GO:0030010	establishment of cell polarity	GO:0007028	cytoplasm organization and biogenesis	0.38	PPI, Exp
GO:0000122	negative regulation of transcription from RNA polymerase II promoter	GO:0000288	mRNA catabolism, deadenylylation-dependent decay	0.54	PPI, GI
GO:0031497	chromatin assembly	GO:0006260	DNA replication	0.62	PPI, GI, Exp
GO:0006269	DNA replication, synthesis of RNA primer	GO:0006272	leading strand elongation	0.83	PPI, GI, Exp

**Figure 1:**
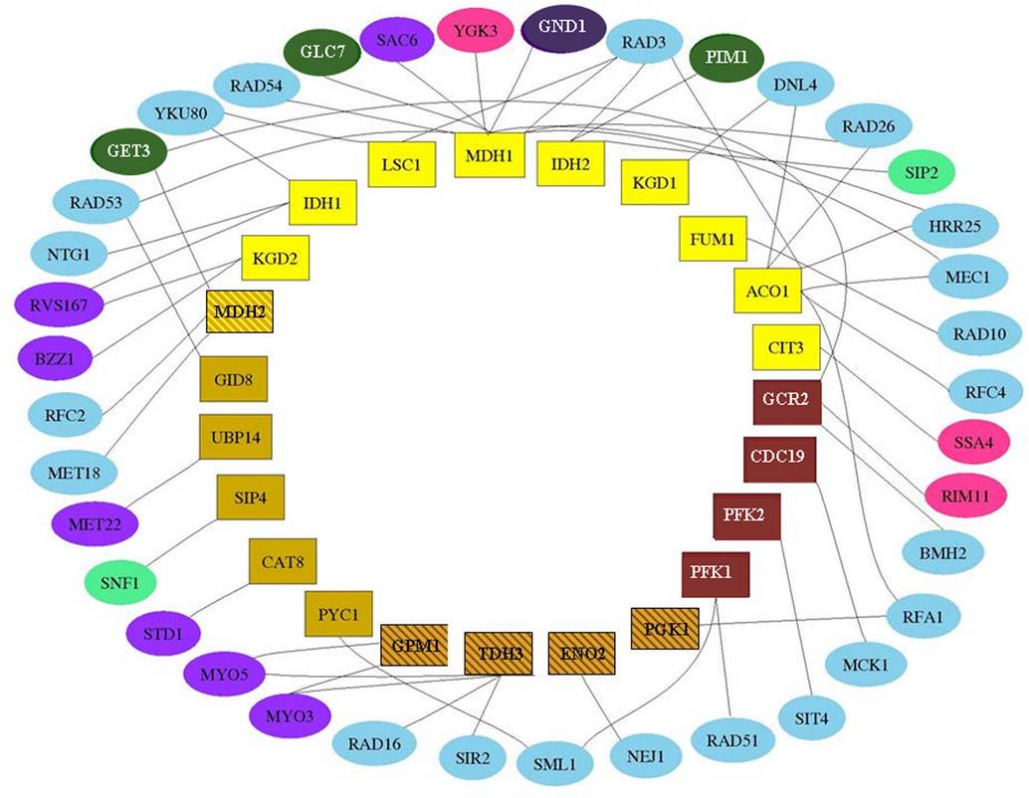
Proteins that participate in “main pathways of carbohydrate metabolism” (rectangular nodes) and interact with proteins that participate in “response to stress” (elliptic nodes). The colors of the nodes represent the sub-term annotation: rectangular nodes correspond to: “tricarboxylic acid cycle intermediate metabolism” (yellow), “gluconeogenesis” (orange) and “glycolysis” (dark brown). Elliptic nodes correspond to “response to-”: “DNA damage stimulus” (bright blue), “osmotic stress” (dark purple), “heat” (dark green), “starvation” (bright green), “oxidative stress” (dark blue). 3 genes are not annotated to any process more specific than “response to stress' (pink). A total of 62 proteins are annotated with “main pathways of carbohydrate metabolism”, and interact with 190 proteins that are not annotated with “main pathways of carbohydrate metabolism”. Of the latter, 38 are annotated with “response to stress”. The total number of proteins annotated with “response to stress” is 337. Consequently, the PPI-link between the two processes is predicted (p-value: 1.47e^−5^).

Some of the other PPI-links are less intuitive. For example, the PPI-link of “main pathways of carbohydrate metabolism” (GO:0006092) to the process “response to stress” (GO:0006950) (semantic similarity 0). This link is of particular interest, since it was recently reported that upon oxidative stress the regulator Stb5 shunts carbohydrate metabolism from glycolysis to the pentose phosphate pathway [11]. Interestingly, Stb5 is currently not annotated to “response to stress” or to “main pathways of carbohydrate metabolism” and therefore it took no part in the prediction of this link. Figure 1 presents the proteins that participate in one of these two processes and interact with each other. Additional less intuitive links are given in the next section.

Some links clearly enrich the universal GO structure with links that are unique to the organism at hand. For example, a link between “hyperosmotic response” (GO:0006972) and “regulation of MAPK activity” (GO:0043405) (shown in details in the Supplement: Text S1 and Figure S1) appears only in the yeast PPI-PLN and not in the PPI-PLNs we constructed for other organisms. Indeed, utilizing the MAPK cascade to regulate the response to hyperosmotic shock is not a universal mechanism, but a known one for the *S. cerevisiae*.

All linked processes are accessible via our site: http://www.cs.bgu.ac.il/~dotna/ProcssLinkageNetworks.

### Comparison of PPI-linkage, expression-linkage and GI-linkage of processes in the yeast Saccharomyces cerevisiae

In order to gain a broader perspective on linked processes we constructed two additional linkage-networks. The first was an expression-PLN, derived from a transcriptional correlation network assembled using expression levels of the yeast genes measured along a cell-cycle [12]. The transcriptional correlation network contains an edge connecting two genes if their expression profiles are highly correlated (see Methods section). The second was a GI-PLN, based on genetic-interaction data. Two genes are called genetically interacting if their mutations have a combined effect not exhibited by either mutation alone (see Methods section).

A total of 545 processes appear in all three networks. The intersection of the three networks (the number of common edges) is many times larger than the size expected at random (see Methods section and Supplement: Text S3 and Figure S3), indicating that “linked processes” tend to physically interact, be co-expressed and genetically interact with each other all at once. The section on GI-prediction shows how this significant co-occurrence of links can be used in the prediction of new genetic interactions.

Table 1 presents examples of links that occur in at least two PLNs, some of which carry an element of surprise. One of these is the link between “bud site selection” (GO:0000282) and “ribosomal large subunit biogenesis ” (GO:0042273), and the link between “nuclear mRNA splicing via U2-type spliceosome” (GO:0006374) and “ribosome biogenesis” (GO:0007046). These links are predicted in both the PPI-PLN and the expression-PLN. The semantic similarities between these linked processes are 0.27 and 0.07, respectively. Finally, there are a considerable number of processes for which the links to other processes vary with the underlying input graph, indicating that the different linkage networks provide non-redundant information.

One example of the different links can be found for the process “protein ubiquitination” (GO:0016567). This process is PPI-linked mainly to processes related to protein catabolism, while it is expression-linked mainly to processes related to rRNA-processing, and GI-linked mainly to cell cycle related processes. Given the role of ubiquitination in protein degradation, it is reasonable that proteins participating in the ubiquitination process will physically interact with proteins participating in the degradation processes and the regulation of this degradation. There are multiple lines of evidence explaining the expression-linkage between protein-ubiquitination and rRNA processing. At the highest level of resolution, transcription, translation and degradation are processes that are expected to be correlated in cell cycle gene expression data. At the more specific level the main ribosomal protein, S27a is synthesized as a C-terminal extension of ubiquitin. The synthesis of ribosomal proteins as extensions of ubiquitin promotes their incorporation into nascent ribosomes by a transient metabolic stabilization and is required for efficient ribosome biogenesis [13]. Moreover, it has recently been shown that complexes associated with pre-rRNA processing factors are ubiquitinated [14].

**Figure 2:**
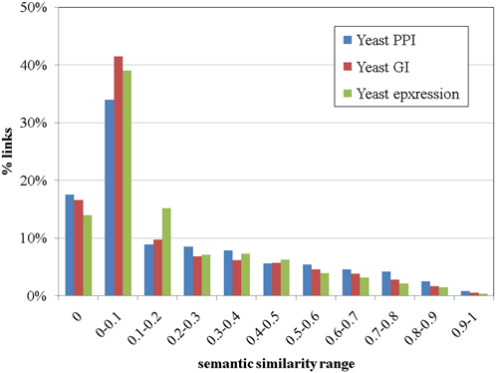
A histogram describing the percentage of process links associated with different semantic similarities. All three networks, show similar distribution, although the number of links is different in the three networks (21,097 links in the PPI-PLN, 48,844 links in the GI-PLN and 4,521 links in the expression-PLN).

Although obtained from different underlying input graphs, all three networks show very similar characteristics in terms of the semantic similarities between the linked processes. Figure 2 shows the distribution of these links as a function of the semantic similarity between the linked processes. In all three networks more than half of the links connect processes that are very distant from each other in the ontology (semantic similarity below 0.1). The reason for that is that our definition of a link specifically excludes the case of a trivial link that can be deduced directly from the ontology, or from the fact that genes have multiple annotations.

We conclude this section with the observation that many of the genetically interacting gene pairs participate in PPI-linked processes. The chance for a pair of *S. cerevisiae* genes with known biological process to participate in specific processes that are PPI-linked is 24% (we refer to a process as specific when at most 200 of the *S. cerevisiae* annotated genes participate in it). This probability increases to 59%, once it is known that the two genes were found to genetically interact with each other.

### Improved functional prediction using linked processes

Functional annotation of currently unannotated proteins is a significant problem, since 35–55% of all newly sequenced genes have no current functional assignment. When a gene without known function is associated with some genes that have a function in common, then it is natural to hypothesize that the same function can be attributed to that gene. This principle is often referred to as “guilt by association”. Two of the most popular associations used for functional prediction, are PPI and transcriptional co-expression. Many algorithms based on PPI-data or expression-data have been proposed in the past few years [15]–[21]. Typically, when the function of a protein *G1* is to be predicted, the algorithm takes into account each protein *G2* which was judged to have something in common with *G1* (*e.g.* it interacts with *G1*, or has a similar expression profile). If *G2* is known to participate in process *t1*, then the probability of predicting *G1* as annotated to *t1* increases. Furthermore, such algorithms utilize the relations between the terms derived from the Gene Ontology: if process *t1* is a descendant of process *t2* in the ontology, then following the “true path rule” *G2* is also annotated with *t2*. Consequently, the probability of predicting *G1* as annotated to *t2* is also increased. In view of the fact that process-links constitute additional relations between processes, we integrated them into functional prediction algorithms. We show here that this integration improves the prediction abilities of the algorithms.

### PPI-based application

In order to evaluate the improvement in the prediction abilities of the standard guilt-by-association method, as described for protein-protein interaction networks [18], [22]–[23], we compared the precision and recall obtained by applying this prediction algorithm to the PfPI network constructed for the *D. melanogaster*, with and without the integration of the PPI-linked processes (IMV and MV, respectively). Both algorithms predict the function of a protein using the known functions of the proteins that are connected to it in the input graph. In the original MV algorithm (simple guilt-by-association), only neighbors that participate in the process under consideration serve as positive witnesses: for a particular GO term *t*, the algorithm sets the state of a protein to +1 if the protein is annotated with *t* and to −1 if the protein is annotated with a different function (in the same GO hierarchy). Our IMV algorithm integrates PPI-linked processes into the prediction by adjoining as positive witnesses also those neighbors that participate in processes that are PPI-linked to the predicted process: for a particular GO term *t*, the state of a protein is set to +1, if the protein is annotated with *t* **or** if the protein is annotated with *t'* and *t'* is linked to *t*. Otherwise the state of the annotated protein is set to −1. Both algorithms are fully described in the Methods section.

**Figure 3:**
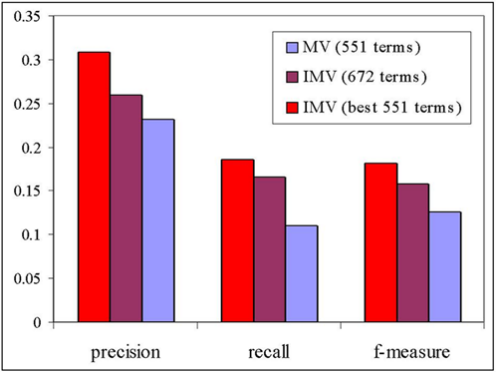
A comparison between the average precision, average recall and average F-measurement obtained by the MV and the IMV methods (for D. melanogaster). For each term the activation threshold was chosen to yield the best F-measurement. The average was taken over those GO-terms for which at least one True Positive was found. There are 551 such GO-terms when using the MV method, and 672 such GO-terms using the IMV method.

Figure 3 summarizes the cross-validation performances of the two prediction procedures (see Methods). The activation threshold was set individually for each term as the value between 0 and 15 that yielded the highest F-measurement (harmonic mean of the precision and the recall) for that term. The average is taken over those GO-terms for which at least one True Positive was found in the cross-validation procedure.

The MV algorithm was able to make predictions for 551 processes. The IMV algorithm made those same predications, of course, but the integration of process linkage into the prediction algorithm, generated predictions for 121 additional GO-processes. When the average precision and average recall of the IMV method were computed for all 672 predictable terms, they were higher by 12% and 51% respectively. By taking the average only over 551 GO-terms, the average precision and average recall increased by 33% and 69% respectively.

**Figure 4:**
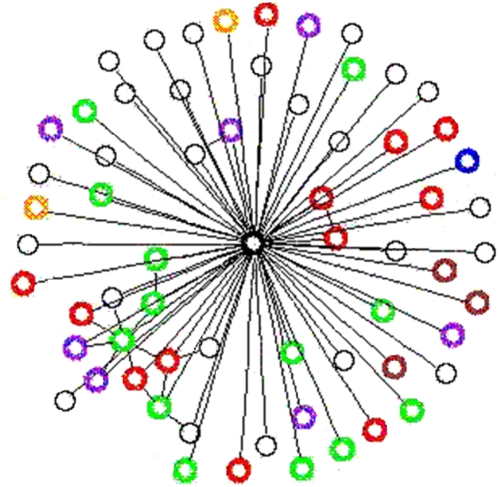
Prediction of “larval development” annotation for the gene CG10701. The sub-network of the D. melanogaster PPI-network containing the genes CG10701, in the middle, and its direct neighbors. Neighbors that currently lack process annotations are black. Neighbors that are annotated with the GO-process “larval development” are blue. Neighbors that are annotated only with processes which were not found to have a PPI-link to “larval development” are red. The other neighbors are annotated with at least one process that was found to have a PPI-link to the predicted process. Purple: cell organization and biogenesis; Green: cell communication; Brown: locomotory behavior; Orange: morphogenesis of an epithelium.

As an example of the heightened predictive power of the IMV algorithm observed in the cross-validation experiment consider the gene CG10701 which is known to have the annotation “larval development” (figure 4). The gene has 63 direct neighbors in the PPI network, of which 25 currently lack functional annotation, one is annotated with “larval development” (GO:0002164) and 25 are annotated with the processes “cell organization and biogenesis” (GO:0016043), “cell communication” (GO:0007154), “locomotory behavior” (GO:0007626), and “morphogenesis of an epithelium” (GO:0002009). The latter processes are all PPI-linked to the process “larval development”. The remaining 12 neighbor proteins are annotated to different processes. Therefore the MV algorithm does not predict that gene CG10701 is annotated with GO:0002164, whereas the IMV algorithm correctly does.

Expression-linked processes can be similarly integrated into expression-based prediction procedures. The resulting functional prediction method for *S. cerevisiae* genes, using cell-cycle expression data [12], made positive predictions for 75 additional GO-terms, increasing the number of predictable GO-terms from 242 to 347. When measuring the average precision, average recall and average F-measurement over 242 GO-terms, we observed an increase in the F-measurement (of 7%, from 0.3 to 0.32), a decrease in the precision (of 10%, from 0.42 to 0.38) and an increase in the recall (of 47%, from 0.25 to 0.37).

This integration also considerably increases the number of new predictions. The MV predicts 5,182 functional annotations for proteins that currently have no annotation, and 5,305 additional functional annotations for annotated proteins. The IMV predicts 9,246 functional annotations for proteins that currently lack one, and 18,437 additional functional annotations for proteins that are currently annotated (see Methods section).

### Improving genetic-interaction predictions using linked processes

**Figure 5:**
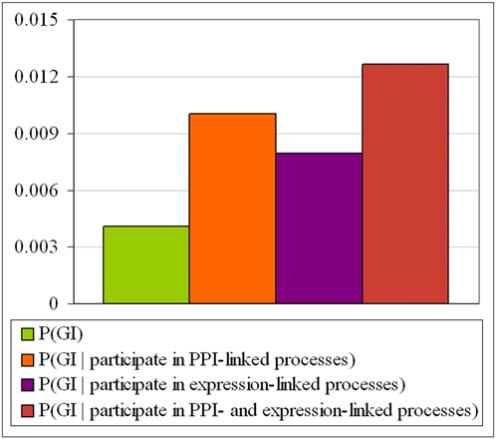
The probability for two genes with known specific biological process (S. cerevisiae) to genetically interact. (i) without prior knowledge; given that the genes participate in (ii) PPI-linked processes, (iii) expression-linked processes and (iv) processes that are both PPI-linked and expression-linked. The total number of genetic interaction that was considered here is 15,228.

PPI-linked processes and expression-linked processes may also useful for the prediction of genetic-interactions. Wong *et al.* used various characteristics of pairs of genes in order to predict genetic interactions [24]. We have established that the probability of two *S. cerevisiae* genes to genetically interact is significantly increased once it is known that the two genes participate in PPI-linked processes or expression-linked processes, by 2.4 and 1.9 times, respectively The probability increases three-fold if it is known that the processes are both PPI-linked and expression-linked (figure 5).

For this calculation only the known *specific* process annotations were considered, in the sense that at most 200 genes are known to participate in such a process.

Similar results were obtained when predicting genetic interactions in *D. melanogaster*, based on PPI-linked processes. We therefore suggest combining this criterion in GI-prediction algorithms.

## Discussion

Proteins in living cells are hypothesized to function as parts of a hierarchy of organized modules controlled by biological processes or pathways. There are significant and important relations between these modules at different levels of biological organization.

At the lowest level biological processes are defined by their parts that include the proteins and their interacting partners. At a slightly higher level processes are defined by regulatory modules that control the condition specific activation of the basic modules. There are also many examples of crosstalk, the sharing of signal components between different signaling pathways, and recently an edge Ontology has been put forth as a platform to examine crosstalk [25]. This work suggests a broader, multi-scale perspective for studying biological processes, which considers relationships between processes in the form of a significantly enriched web of cross-process physical interactions, co-expression and genetic interactions generating PPI-linked, expression-linked and GI-linked process networks respectively. It is natural for these links to be asymmetric, since processes in the living cell influence each other in an asymmetric fashion, occurring sequentially, one regulating another etc. We elaborate on the a-symmetry of the links in the Supplement (Text S1 and Figure S1). We emphasize that our method predicts the existence of links, but it does not aim to explain the underlying causal reason for these links or for their directionality.

The validity of the predicted interactions between processes is supported by our finding that the likelihood of a genetic interaction between proteins is significantly increased if the proteins belong to two different “linked processes”. The fact that some processes tend to genetically-interact with each other, may shed light on the evolving field of genetic interactions. Our findings are complementary to the hypothesis put forth by Kelley and Ideker [26] that genetic interactions are more likely to bridge redundant or complementary processes than to combine additively within the same process. We discuss the difference between the two approaches, both leading to this conclusion, in the Supplement (Text S4). Segre *et al.* looked at genetic interactions between functional units and found that these interactions tend to be either exclusively buffering or exclusively aggravating [27].

Linked processes were shown to improve both the coverage and the accuracy of functional prediction methodologies. Some of the edges between processes were detected in multiple type networks. We also found many of the process links to be evolutionarily conserved across species.

Needless to say, the specific methodology described in this paper deserves a more thorough investigation and is likely to evolve in the future. For instance, a natural alternative for our “enriched interactions” between processes would be a method that measures which additional processes will increase the accuracy of predicting some behavioral aspect of a given process. Information theoretical analysis in ontologies, based on measures such as “semantic similarity” [9], [28]–[31], is commonly used to establish relationships between processes. However such an approach documents known connections between proteins that are involved in two or more processes. We deliberately excluded these from our analysis since our study is complementary to “semantic similarity”. The edges in the GO-hierarchy represent “is a” and “part of” relations. They do not represent other relations, such as complementariness. In contrast, we link processes based on “connectivity” established outside the ontology itself. This approach uncovers links that are not explicit in the hierarchy, and hence are undetectable using semantic similarity measures. Moreover, the definition of semantic similarity presupposes a hierarchy structure, whereas linked processes can be defined for any set of annotation terms (e.g. KEGG).

The most provocative conclusion one can derive from the analysis provided in this paper is the need to re-examine our current approaches for organizing biological knowledge. Ontologies have been the most prominent principled methodologies to catalogue biological knowledge. However, drawing upon one of the lessons learned during the evolution of the WWW, in which a self-evolving network organization largely replaced the initial attempts to organize the web as an ontology (e.g. the early days of Yahoo), we hypothesize that biological ontologies will benefit from a complementary multi-scale network supported data structures that provide additional useful connections to organize complex multi-dimensional data.

For now, the framework of linked processes described in this paper constitutes a promising new direction towards understanding the higher levels of organization of the cell as a system as well as improving our ability to predict which proteins are involved in specific biological processes at the lower levels.

## Materials and Methods

### Definition of linked processes

The definition of linked biological processes is based on an input (undirected) graph whose nodes correspond to proteins and whose edges correspond to some form of functional connection between the proteins, such as protein-protein interaction, co-expression or genetic interaction. Such an annotated input graph is often referred to as a functional linkage graph [18]–[19]. We consider only those proteins that are annotated to at least one term. It is the purpose of a link from process term *i* to process term *j* to indicate that proteins annotated with term *i* tend to interact with proteins annotated with *j* to a greater extent than expected by chance. The statistical enrichment of *j* is computed with respect to the set of proteins that are connected to the proteins annotated with *i* in the input graph, excluding proteins that participate in both *i* and *j*. This exclusion prevents the creation of an abundance of hypothesized links that could result from proteins that have multiple annotations and interact with each other, or from proteins that are connected in the GO-hierarchy in a “is a” relation. However, a process *i* that is linked to a process *j*, where *i* “is a” *j*, remains of interest since it arises when proteins that are annotated with process *i* interact with many proteins that are annotated with process *j* but not with process *i*.

Here is the formal definition:

Let *Ni* be the set of nodes that are annotated with process *i* (and possibly with other processes as well).
Let *NBi* be the set of nodes that are connected to at least one node that is annotated with process *i* but that are not annotated with process *i* themselves:
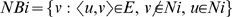

With these definitions, the probability of finding at random, among the set of proteins that are connected to at least one node that is annotated with process *i* but that are not annotated with *i* themselves, a number of proteins equal to or bigger than the number of proteins that were found actually to be annotated with process *j*, |*NBi*∩*Nj*| is
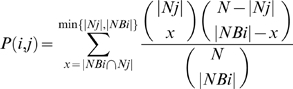



We will say that process *i* is linked to process *j*, if process *j* is statistically enriched in *NBi*, in the sense that *P(i,j)*<*threshold*. The threshold was set at 0.001, in order to filter out false positives at a level that is satisfactory according to a straightforward FDR calculation and statistically significant according to two randomization procedures (see next section).

An illustration of the determination of a link and a discussion of some of its properties are given in the Supplement (Text S1 and Figure S1).

### PPI-linked processes

We call *PPI-linked processes* those linked processes obtained when the input graph is a PPI-network.

We computed 3 sets of PPI-linked terms. Since the linkage of processes is defined using a network in which all nodes are annotated, we excluded the unannotated nodes and the induced edges, to create a fully annotated subnetwork.

The first set resulted from the PPI data for *S. cerevisiae*, obtained from the Database of Interacting Proteins (DIP) [32] (version 04 Sep 2005), available online at http://dip.doe-mbi.ucla.edu. This network currently contains 4,765 nodes (proteins) connected by 15,518 edges (interactions). The proteins in this network were annotated by mapping biological process annotations for *S. cerevisiae* (Revision: 1.1190, 30 Sep 2005) from the GO consortium [33].

The second set was determined from the PPI data for *D. melanogaster* [34]–[35]. The combined network contains 7,336 nodes connected by 21,708 edges. The proteins in this network were annotated by mapping biological process annotations for *D. melanogaster* (Revision: 1.60, 23 Jul 2005) from the GO consortium.

The third was based on the PPI data for *H. sapiens*, obtained from OPHID [36], available online at http://ophid.utoronto.ca/ophid/. This network currently contains 9,098 nodes connected by 41,376 edges. The proteins in this network were annotated by mapping biological process annotations for *H. sapiens* (Revision: 1.18, 8 Oct 2005) from the GO consortium.

Since the GO annotations form a directed acyclic graph (DAG), many of the annotations of the gene products are implicit. Therefore in our analysis every protein annotated with process *t*, was annotated also with all processes more general than *t* (all ancestors of *t* in the DAG).

After the exclusion of the unannotated proteins, the *S. cerevisiae* network contains 3,429 nodes connected by 12,406 edges (0.1% of the possible edges), the *D. melanogaster* network contains 4,187 nodes connected by 7,754 edges (0.044% of the possible edges) and the *H. sapiens* network contains 5,601 nodes connected by 21,702 edges (0.07% of the possible edges).

**Figure 6:**
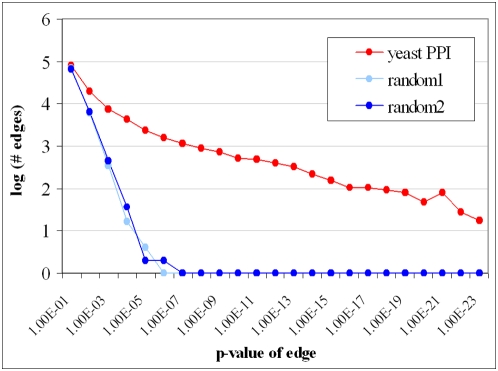
Log-log plot of the number of pairs of linked processes, as a function of the p-value of the link (PPI-Links, S. cerevisiae). Both types of random annotations yield far fewer pairs with very low p-values than the actual network.

To assess statistical significance we compiled a histogram of pairs of linked processes, corresponding to two types of random annotations for the proteins derived from the *S. cerevisiae* network scaffold. In the first type of random annotation, named *random1*, the original *sets* of protein annotations were randomly redistributed among the proteins. This can be viewed equivalently as the procedure of assigning proteins randomly to the vertices of the given PPI-network. For the second type of random annotation, named *random2*, a new PPI-network was constructed by placing an edge between two proteins with probability equal to the fraction of edges in the original graph. The results of the comparisons between the obtained numbers of edges are summarized in figure 6. The number of pairs of terms that are associated with p-value of 0.01–0.1 (above our threshold) is very similar to the number of pairs that are associated with this p-value in each of the two random annotation procedures. However, the number of pairs of linked processes that are associated with very low p-value is negligible in each of the random annotation procedures, while the number of pairs of linked processes decreases slowly in the real *S. cerevisiae* PPI-linked network. Note that the two random annotation procedures yield very similar distributions of p-values.

### Expression-linked processes

We call a pair of processes *expression-linked processes* if they are linked when the input graph is based on expression data. This graph, often referred to as a relevance network [37], has nodes representing genes and edges representing co-expression between genes, in the sense that according to some metric the similarity of the expression patterns is above some threshold.

To construct the relevance network for *S. cerevisiae*, we used a data-set comprising the expression levels of 5595 of the predicted *S. cerevisiae* genes, in 42 different stages of the cell-cycle [12], available online at: http://genome-www.stanford.edu/cellcycle/data/rawdata. The relevance network contained an edge between two genes if the absolute Pearson correlation between their expression profiles is at least 0.85; all singleton nodes were deleted. Next, the genes in this network were annotated by mapping biological process annotations for *S. cerevisiae* (Revision: 1.1190, 30 Sep 2005) from the GO consortium [33]. As before, each gene product annotated with term *t*, was also annotated with all terms that are more general than *t*, and all unannotated nodes were deleted, to create a fully annotated sub-network. The resulting network consists of 997 nodes connected by 4,168 edges (0.42% of the possible edges).

### Genetic interaction data

We call *GI-linked processes* those linked processes obtained when the input graph is constructed on the basis of genetic interactions. A GI-network was constructed for *S. cerevisiae*. In this network nodes represent genes and edges represent genetic interactions.

The list of genetic interactions for *S. cerevisiae* (dosage growth defect, dosage lethality, dosage rescue, phenotypic enhancement, phenotypic suppression, synthetic growth defect, synthetic lethality and synthetic rescue)., was obtained from BioGRID [38], the general repository for interaction datasets, (version 2.0.20), available online at http://www.thebiogrid.org. The biological process annotations for *S. cerevisiae* (Revision: 1.1190, 30 Sep 2005) from the GO consortium were used to annotate the genes.

The resulting network contained 2,904 nodes and 21,942 edges (0.52% of the possible edges).

### Calculating FDR

In some instances of the present analyses, we generate a multiplicity of hypotheses. We compute the FDR (q-value) as follows:

Let *R* denote the number of hypotheses rejected by a procedure.
Let *V* denote the number of true null hypotheses erroneously rejected (type I error).
*q* denotes *V*/*R* when *R* > 0 and 0 otherwise.

In our context, *V* is the number of expected false-positive predicted links between GO-terms (at a given p-value threshold p, *V* = p×number of hypotheses) and *R* is the number of predicted links at the current *p*-value threshold.

Of the 21,097 directed edges of the Process Interaction Network constructed for the *S. cerevisiae*, 1,347 are expected to be false discoveries (0.001 *p*-value×1,161 nodes×1,160 nodes = 1,347). Therefore, calculation of the FDR *q*-value for a *p*-value of 0.001 yields a *q*-value of 0.06, or 6% (*q*-value = 1,347/21,097 = 0.06).

### Semantic similarity measurements

We use the measurement suggested by Lin [9].
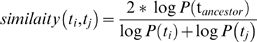



Where *P(t_i_)* is the probability of a gene to be annotated with term *t_i_* (namely: the number of genes annotated with *t_i_* divided by the total number of genes). *t_ancestor_* is the most specific common ancestor-term of *t_i_* and *t_j_* (the one with the least number of genes annotated to it).

This similarity measure has the following desirable properties:
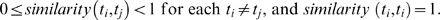



### Statistical significance of the intersection between different sets of linked processes

To determine the statistical significance of the size of the intersection between three sets of linked process, we computed the p-value of obtaining an intersection of this size or larger when three sets of the given sizes are drawn at random.

### Functional prediction – simple guilt-by-association

The specific implementation we use relies on the procedure described in Karaoz *et al.* where a separate network is constructed for each GO term^19^. For a particular GO term *t*, the algorithm sets the state of a protein to +1 if the protein is annotated with *t* and to −1 if the protein is annotated with a different function (in the same GO hierarchy). Next, the algorithm repeatedly selects an unannotated protein *i*, representing a hypothetical protein, and sets its state *s_i_* to −1 or +1 using the following activation rule
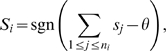
where *n_i_* is the number of neighbors of protein *i*, *s_j_* is the state of neighbor *j*, and *θ* is an “activation threshold”. The right hand side of this equation computes the sum of the states of the neighbors of node *i* and sets the state of node *i* to +1 if the sum is 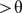, and to −1 otherwise. For that reason, this algorithm is referred to as a “Majority-Vote algorithm”. In our implementation the activation threshold was chosen for each GO-term independently and set at that value, between 0 and 15, that yielded the best F-measurement score in cross-validation tests.

Finally, the algorithm assigns the putative annotation *t* to all hypothetical proteins whose state was set to +1.

The algorithm is applied only to those GO-terms for which the cross validation procedure yielded at least one positive prediction.

### Functional prediction – Improving the algorithm by integrating linked processes

Similarly to the original algorithm, a distinct network is constructed for each GO term. For a particular GO term *t*, the state of a protein is set to +1, if the protein is annotated with *t* **or** if the protein is annotated with *t'* **and** *t'* is linked to *t*. Otherwise the state of the annotated protein is set to −1. Next, the hypothetical proteins are assigned a state of +1 or −1 in the same way as before. Finally, the putative annotation *t* is assigned to each hypothetical protein whose state was set to +1 provided at least one of the neighbors of the hypothetical protein is annotated with *t*. The activation threshold was selected for each GO-term separately, as described for the simple guilt-by-association procedure. Again, predictions were made only for those GO-terms for which at least one positive prediction was generated in the cross-validation test.

### Functional prediction – Assessment

The prediction performances of the different procedures were measured by cross-validation. Separately for each GO-process term *t_i_*, three measurements, precision *p_i_*, recall *r_i_* and F-measurement *f_i_* were computed. These measurements are based on counting true positives (*TP_i_*), the number of genes correctly assigned; false positives (*FP_i_*), the number of genes incorrectly assigned; false negatives (*FN_i_*) the number of genes incorrectly not assigned. The precision and recall for term *t_i_* are defined as follows:
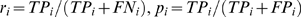



F-measurement, the harmonic mean of the precision and the recall is defined as follows: f_i_ = 2_*_ r_i*_ p_i_/(r_i_+p_i_).

Let *T* be a set of GO-processes. The overall precision, recall and F-measurement are then:
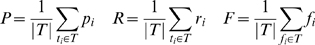



## Supporting Information

Text S1 An illustration of link determination
(0.03 MB DOC)

Text S2 Degree distribution in the S. cerevisiae PPI-PLN
(0.03 MB DOC)

Text S3 Comparison of PPI-linkage, expression-linkage and GI-linkage of processes in the yeast Saccharomyces cerevisiae.
(0.03 MB DOC)

Text S4 Genetic interactions are more likely to bridge redundant or complementary processes: The differences between the approaches.
(0.02 MB DOC)

Figure S1 A) The yeast PPI-subnetwork consisting of only those genes that are annotated with “regulation of MAPK activity” (yellow nodes) or “hyperosmotic response” (black nodes), and their neighbors. B,C) In order to test whether there is an edge linking term t1 to term t2, one needs to consider the genes that are annotated with t1 (green nodes), those of their neighbors that are not annotated with t1 (blue nodes), the genes that are annotated with t2 but are not annotated with t1 (red nodes) and the intersection of the latter two (purple nodes). B) The test for an edge linking “hyperosmotic response” to “regulation of MAPK activity” is positive. C) The test for an edge linking “regulation of MAPK activity” to “hyperosmotic response” is negative.
(0.43 MB TIF)

Figure S2 Complementary Cumulative Distribution of the in- and out-degrees in the PLN obtained for yeast. A) In-degree; B) Out-degree.
(0.17 MB TIF)

Figure S3 Venn diagram representation of the sizes of the intersections between the sets of pairs of PPI-linked, expression-linked and GI-linked processes. Each set contains only linkages between processes that appear in all three types of linkage networks.
(0.14 MB TIF)
