# Supplementary material for: Biological Process Linkage Networks
Source: PLoS One. 2009 Apr 23;4(4):e5313. doi: 10.1371/journal.pone.0005313 (PMC2669181; doi:10.1371/journal.pone.0005313)
Supplement: Text S1 — An illustration of link determination (0.03 MB DOC) [file pone.0005313.s001.doc]

Figure S1 illustrates a situation in which an edge between two terms is created in only one direction. In Figure S1 B, an edge from the process "hyperosmotic response" (GO:0006972) to "regulation of MAPK activity" (GO:0043405) is inferred: The 7 genes that are annotated with "hyperosmotic response" interact with 18 other genes among which there are 4 genes that are annotated with "regulation of MAPK activity" and that are not annotated with "hyperosmotic response". The probability to obtain these numbers by chance is 8*e-9, a p-value below our threshold. In Figure S1 C the existence of the reverse edge is examined. The 6 genes that are annotated with "regulation of MAPK activity" interact with 35 other genes among which there are 2 genes that are annotated with "hyperosmotic response" and that are not annotated with "regulation of MAPK activity". The probability of obtaining these numbers by chance is 0.002, a p-value which is above our threshold. In this case the link reflects a cause-and-effect relationship: in response to hyperosmotic stress *S. cerevisiae* heavily regulates the MAPK cascade. The fact that the two processes are distant from each other in the ontology indicates that this cause-and-effect is a mechanism that is not universal. To summarize, factors influencing the formation of an edge in only one direction are the differences in the total number of interacting partners that the processes have, as well as the pattern of interactions between the two processes.
