# Supplementary material for: Biological Process Linkage Networks
Source: PLoS One. 2009 Apr 23;4(4):e5313. doi: 10.1371/journal.pone.0005313 (PMC2669181; doi:10.1371/journal.pone.0005313)
Supplement: Text S2 — Degree distribution in the S. cerevisiae PPI-PLN (0.03 MB DOC) [file pone.0005313.s002.doc]

An interesting observation is that the distribution of the in-degrees is very different from the distribution of the out-degrees. 1,081 of the 1,161 nodes have out-edges, whereas only 732 of the nodes have in-edges. The highest in-degree is much higher than the highest out-degree (245 and 111 respectively). The imbalance between the in- and the out-degree can be attributed to the fact that the edges of the PLN are directed.

The process terms with the largest number of outgoing edges are all related to cell organization, while the processes that have the largest number of incoming edges are all cell-death related.

The difference in the distributions of the in- and the out-degrees is observed also in the first of the two random annotation procedures described in the previous section, random1, in which the proteins were randomly reassigned to the nodes of the original PPI graph. The in- and out-degrees were balanced, however, when the second randomization procedure, *random2*, was employed. The topology of the graph generated by *random2* is different from the topology of the graph generated by *random1*. It can therefore be concluded that the imbalance between the in- and the out-degree is a consequence of the scale-free topology of the PPI-network. No correlation was detected between the in-degree, or the out-degree, of a GO-term and its frequency or the number of neighbors that a term has (The number of proteins that are not annotated with a term but whose neighbors in the PPI-network are annotated with that term). Nor was a correlation between the in-degree and the out-degree of the term-nodes detected.

The underlying (undirected) network consists of 9 connected components: 8 small ones (containing 2-5 nodes) and a large component, containing the vast majority of the processes.

Figure S2 shows a log-plot of the Complementary Cumulative Distribution of in-degrees (Figure S2 A) and the out-degrees (Figure S2 B) for the sub-networks containing only the best {500, 1000, 2500, 5000, 10000} edges, when the edges are sorted according to their p-values. All networks exhibit approximately linear curves, indicative of an exponential distribution. The fast decaying tail of this distribution may be the result of a limited number of interactions between the participating proteins^38-39^ or alternatively it may be caused by the statistical calculation (see Methods section) that defines statistical enrichment with respect to the background annotations.
