# Supplementary material for: Biological Process Linkage Networks
Source: PLoS One. 2009 Apr 23;4(4):e5313. doi: 10.1371/journal.pone.0005313 (PMC2669181; doi:10.1371/journal.pone.0005313)
Supplement: Text S3 — Comparison of PPI-linkage, expression-linkage and GI-linkage of processes in the yeast Saccharomyces cerevisiae. (0.03 MB DOC) [file pone.0005313.s003.doc]

At a p-value of 0.001 there are 21,097 pairs of PPI-linked processes involving 1,161 processes, 4,521 pairs of expression-linked processes involving 634 processes (q-value = 0.088) and 48,844 GI-linked processes involving 1,378 processes (q-value = 0.04).

Only 545 processes appear in all three lists of linked processes. The numbers of pairs of PPI-linked, expression-linked, and GI-linked processes, connecting only these 545 processes, are respectively 12,600 (q-value = 0.02), 4,021 (q-value = 0.07), and 21,737 (q-value = 0.014). All the intersections between the sets of PPI-linked and expression-linked pairs of processes, between PPI-linked and GI-linked pairs of processes, and between expression-linked and GI-linked pairs of processes are many times larger than expected at random – 7.9 times, 3.5 times¸ and 5.6 times respectively (Figure S3). Moreover, the intersection between the three sets is 47 times larger than expected at random. All relevant p-values are vanishingly small.
