# Supplementary material for: Biological Process Linkage Networks
Source: PLoS One. 2009 Apr 23;4(4):e5313. doi: 10.1371/journal.pone.0005313 (PMC2669181; doi:10.1371/journal.pone.0005313)
Supplement: Text S4 — Genetic interactions are more likely to bridge redundant or complementary processes: The differences between the approaches. (0.02 MB DOC) [file pone.0005313.s004.doc]

Genetic interaction between proteins is more likely if the proteins belong to two different “linked processes”. This finding is complementary to the hypothesis put forth by Kelley and Ideker [9], that genetic interactions are more likely to bridge redundant or complementary processes than to combine additively within the same process.

However, our method of prediction, which relies on different links between pre-defined GO-categories, is very different from Kelly and Ideker's one. Their method is based on the degree of connectivity of a network that has two kinds of edges: the first kind represents physical interactions of three types: protein-protein interactions, protein-DNA regulatory interactions and shared-reaction metabolic relationship. The second kind of edges represents genetic interactions (both synthetic-lethal and synthetic-sick). For this network, Kelley and Ideker identified the between-pathway model as the most abundant model**.** They assume this model when two distinct sub-graphs are highly connected by physical-interaction edges, and there are a significant number of genetic-interaction edges connecting these two subgraphs. We emphasize that this definition of between-pathway interactions, does not take into account the known functional annotations of the genetically interacting genes, which serve as a key factor in our approach.
