# Supplementary figures and images for: Biological Process Linkage Networks
Source: PLoS One. 2009 Apr 23;4(4):e5313. doi: 10.1371/journal.pone.0005313 (PMC2669181; doi:10.1371/journal.pone.0005313)

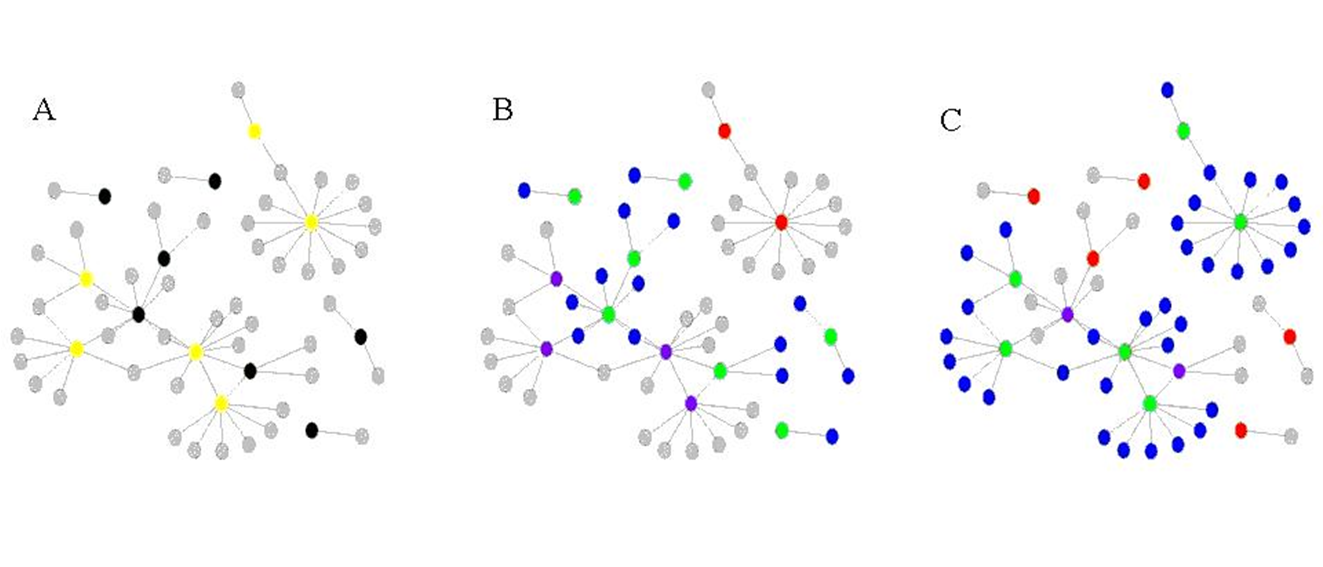

Supplement: Figure S1 — A) The yeast PPI-subnetwork consisting of only those genes that are annotated with “regulation of MAPK activity” (yellow nodes) or “hyperosmotic response” (black nodes), and their neighbors. B,C) In order to test whether there is an edge linking term t1 to term t2, one needs to consider the genes that are annotated with t1 (green nodes), those of their neighbors that are not annotated with t1 (blue nodes), the genes that are annotated with t2 but are not annotated with t1 (red nodes) and the intersection of the latter two (purple nodes). B) The test for an edge linking “hyperosmotic response” to “regulation of MAPK activity” is positive. C) The test for an edge linking “regulation of MAPK activity” to “hyperosmotic response” is negative. (0.43 MB TIF) [file pone.0005313.s005.tif]

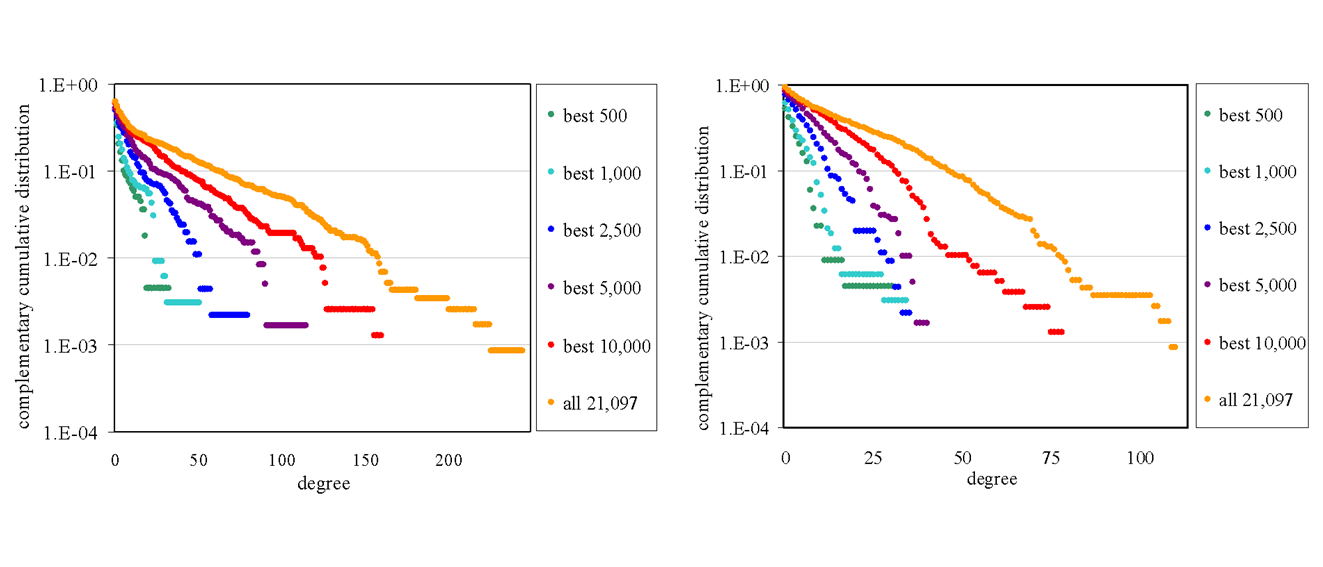

Supplement: Figure S2 — Complementary Cumulative Distribution of the in- and out-degrees in the PLN obtained for yeast. A) In-degree; B) Out-degree. (0.17 MB TIF) [file pone.0005313.s006.tif]

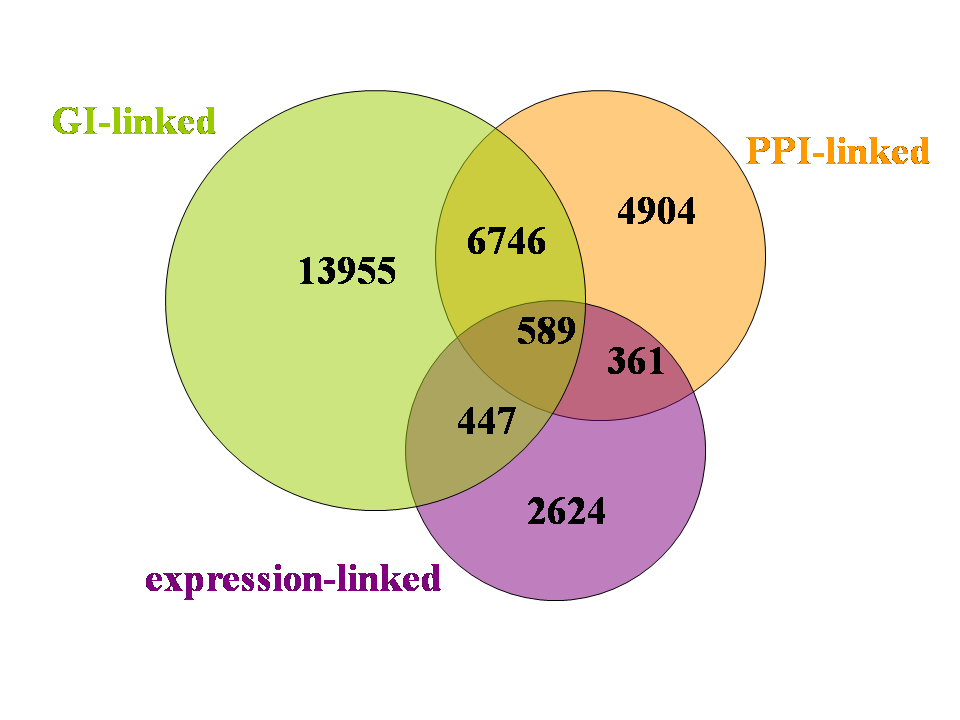

Supplement: Figure S3 — Venn diagram representation of the sizes of the intersections between the sets of pairs of PPI-linked, expression-linked and GI-linked processes. Each set contains only linkages between processes that appear in all three types of linkage networks. (0.14 MB TIF) [file pone.0005313.s007.tif]
